# Comparative Study of Sex Differences in Distal Femur Morphology in Osteoarthritic Knees in a Chinese Population

**DOI:** 10.1371/journal.pone.0089394

**Published:** 2014-02-19

**Authors:** Bo Yang, Jia-Kuo Yu, Zhuo-Zhao Zheng, Zhi-Hua Lu, Ji-Ying Zhang

**Affiliations:** 1 Institute of Sports Medicine, Peking University Third Hospital, Haidian District, Beijing, China; 2 Department of Radiology, Peking University Third Hospital, Haidian District, Beijing, China; Kroto Research Institute, United Kingdom

## Abstract

**Objectives:**

The purpose of this study was to investigate sex differences in resected distal femoral morphology in Chinese osteoarthritic knees.

**Methods:**

The study included 130 osteoarthritic knees in 65 men and 65 women in China. None had anterior femoral osteophyte or serious patellar femoral joint degeneration. The following were measured using computed tomography and analyzed to identify morphological differences according to sex in the resected distal femurs: anterior lateral condylar height (ALCH), anterior medial condylar height (AMCH), and mediolateral (ML) and anteroposterior (AP) dimensions. The ML/AP aspect ratio was calculated.

**Results:**

The average ALCH and AMCH were 8.2±1.8 mm, 3.1±1.5 mm for men and 7.4±1.7 mm, 3.6±1.5 mm for women. There were significant differences between men and women in ALCH values (P = 0.014) but not in AMCH values (P = 0.09). Women had smaller ML/AP aspect ratios than men for a given AP dimension. This indicated that the femoral ML dimension of a prosthesis with a given AP dimension may have overhang in women.

**Conclusions:**

This study suggested that sex differences should be taken into account in the design of femoral prosthesis for Chinese men and women.

## Introduction

To achieve a successful outcome during total knee arthroplasty (TKA) and to reduce complications, it is important that the size and shape of the knee prosthesis matches the morphology of the resected knee [1], [2]. Many studies have shown striking sex differences in distal femoral morphology, and these differences have prompted the design of female-specific femoral components with narrower femoral condyles, thinner anterior flanges, and increased trochlear groove angles that provide a better fit for the anatomical variances in women’s knees [3]–[5]. Compared with standard femoral components, female-specific femoral components can reduce the mediolateral overhang in women undergoing TKA [6], [7]. However, it is not known whether use of female-specific femoral components results in better clinical outcomes in women. Some researchers support using sex-specific femoral components [3], [8], whereas others express doubts that the use of such components is necessary [9]–[11].

Morphological differences have also been reported among some populations that differ in terms of ethnicity. Specifically, Asian populations have different femoral dimensions and morphology than Western populations [12], [13]. However, most currently available commercial knee prostheses are designed based on anthropometric data from Caucasian knees; such prostheses do not necessarily provide the best fit in Asian populations. For instance, with currently used TKA implants, the femoral component tends to show mediolateral overhang [1], [14] or poor clinical results for TKAs performed in Asian populations [15]. These findings, along with the increasing use of TKA in Asian countries [16], [17], indicated that it is critical to investigate knee morphology in Asian populations.

Furthermore, most knees undergoing TKA are different from healthy knees, suggesting that the prosthetic design should be based on the data from diseased knees [18]. Our previous study found that in Chinese subjects with osteoarthritis, the proximal tibias of men and women had the same AP and different ML dimensions; this resulted in a higher ML/AP aspect ratio in men compared to women [19]. Because osteoarthritis affects the shape of the knee, the present study investigated sex differences in the geometric features of the distal femur in Chinese osteoarthritic knees using three-dimensional computed tomography (CT).

## Materials and Methods

### Patients

This study was approved by the institutional review board at the Peking University Third Hospital. Prior to the start of the study, all study subjects gave written informed consent. A total of 130 osteoarthritic knees from 65 Chinese men and 65 Chinese women were included in the study. The mean subject age was 61.4±8.3 years for men and 61.6±7.7 years for women. The mean height was 169.3±4.2 cm for men and 160.0±5.4 cm for women. Patients were excluded if they had a history of femoral fracture or congenital anomaly, if they had diseases that could affect the normal formation of the knee joint, or if the knee had a varus or valgus deformity greater than 15°.

### CT Imaging

A CT scan of each knee was performed using a helical CT scanner (120 kVp, 200 mA, Somatom Sensation 16, Siemens Healthcare, Germany). The patient was placed supine with the knee in a full extended position on the scanner with the patella facing towards the ceiling. The scanning procedure acquired 1-mm CT slices (image size, 512×512 pixels). The CT images were retrieved on the CT workstation (Syngo CT workplace, Germany), and the femur was segmented to construct three-dimensional bone models.

### Measurements

**Figure 1:**
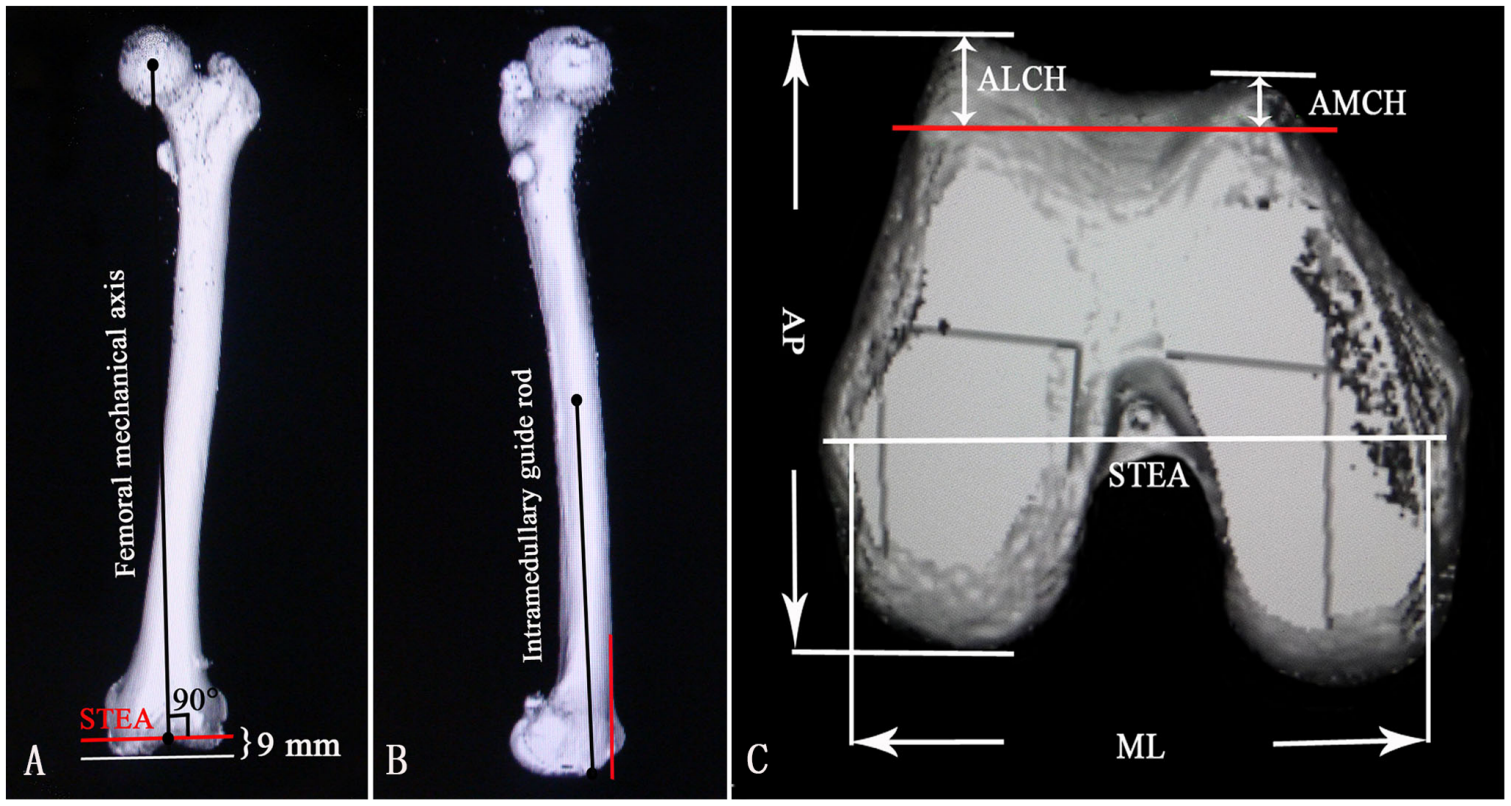
Cuts and measurements of the distal femur on computed tomography (CT) images. (A) A coronal CT image of a femur. The red line indicates the distal femur cut line. (B) A sagittal CT image of a femur. The red line indicates the anterior condyle cut line, which is flush with the anterior femoral cortex. (C) An axial CT image shows the measurement parameters for the distal femur. The red line indicates the anterior condyle cut line.

Using the CT images, a line that connected the center of the femoral head to the center of the distal femur notch was defined as the femoral mechanical axis. A line connecting the medial sulcus of the medial epicondyle and the lateral epicondylar prominence was defined as the surgical transepicondylar axis (STEA). A line connecting the middle point of the femoral shaft to the entry point of the intramedullary guide rod (10 mm above the roof of the intercondylar fossa) was defined as the simulated intramedullary guide rod. The distal femur was cut passed the STEA and perpendicular to the femoral mechanical axis with 9 mm above the lowest point of the medial femoral condyle (Figure 1A). The anterior condyle was cut parallel to the simulated intramedullary guide rod without notching the anterior cortex (Figure 1B). The femoral mediolateral (ML) dimension was defined as the longest ML length of the distal cut femur surface; this line paralleled the STEA. The anteroposterior (AP) dimensions were defined as the longest line drawn perpendicular to the ML line between the most posterior condylar and the anterior trochlear point from the lateral condyle of the femur. The anterior lateral condylar height (ALCH) and the anterior medial condylar height (AMCH) were defined as the maximum thicknesses of the anterior cut at the lateral and medial condyles, respectively (Figure 1C). The femoral aspect ratio of the ML dimension to the AP dimension (ML/AP) was calculated.

### Statistical Analysis

SPSS software version 18.0 (SPSS, Chicago, IL) was used for statistical analysis. The mean and standard deviation of the measured dimensions were calculated. For independent samples, the student’s t-test was used to determine the significance of differences between values for men and women. Linear regression analysis was used to determine correlations for the femur ML and AP dimensions. The differences were considered significant when P<0.05.

## Results

The average ALCH was 8.2±1.8 mm for men and 7.4±1.7 mm for women. There was a significant difference between men and women with regard to ALCH (P = .014). The average AMCH was 3.1±1.5 mm for men and 3.6±1.5 mm for women (P = 0.09). The average ML and AP dimensions and ML/AP aspect ratios were significantly lower in women than in men (P<0.05; Table 1).

**Table 1:** Distal femur dimensions (mm) in Chinese men (n = 65) and women (n = 65).

Parameter	Men	Women	P
Anterior lateral condylar height (ALCH)	8.2±1.8	7.4±1.7	0.014
Anterior medial condylar height (AMCH)	3.1±1.5	3.6±1.5	0.090
Mediolateral length (ML)	79.0±5.0	71.2±4.3	<0.001
Anteroposterior length (AP)	66.8±4.0	61.3±3.3	<0.001
ML/AP aspect ratio	1.18±0.06	1.16±0.05	0.031

**Figure 2:**
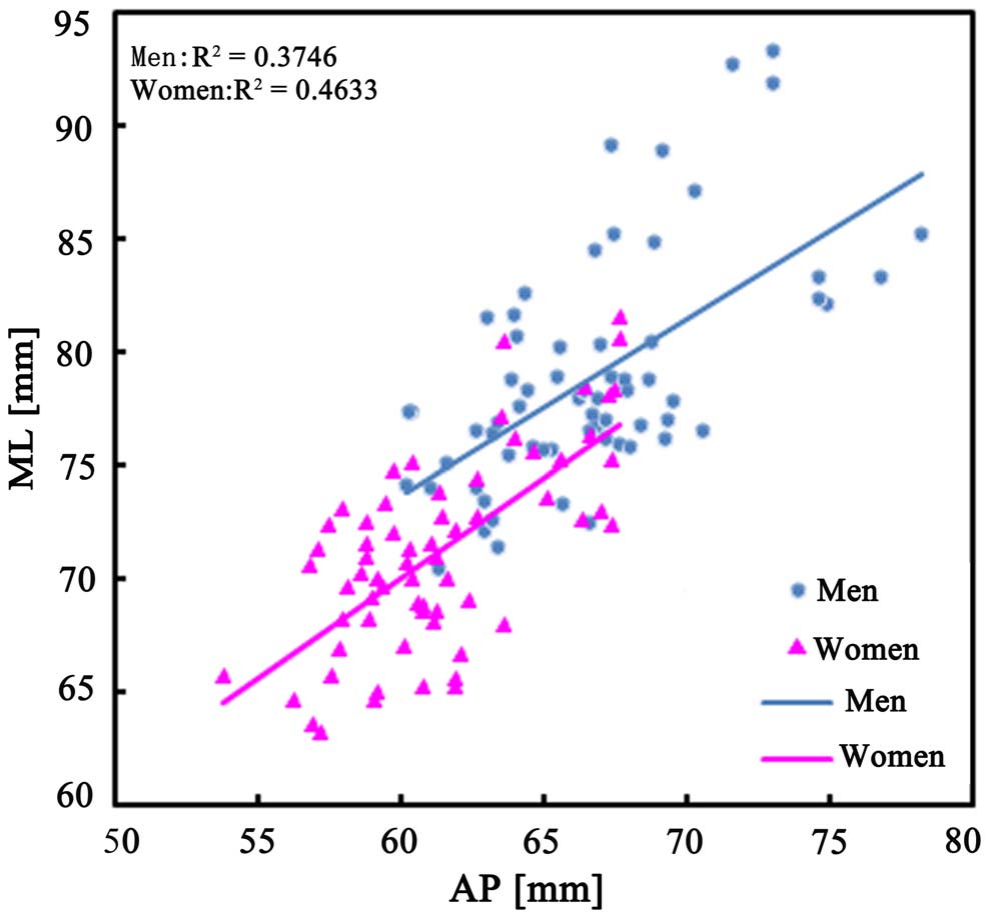
The ML dimensions were plotted against the AP dimensions for men and women.

There was a significant positive correlation between the ML and AP dimensions in both men and women, with the ML dimension increasing as the AP dimension increased. The line fitted to the data for women lies below the one of men, showing that women generally have a smaller ML dimension than men for a given AP dimension, i.e. men have wider knees than women (Figure 2).

**Figure 3:**
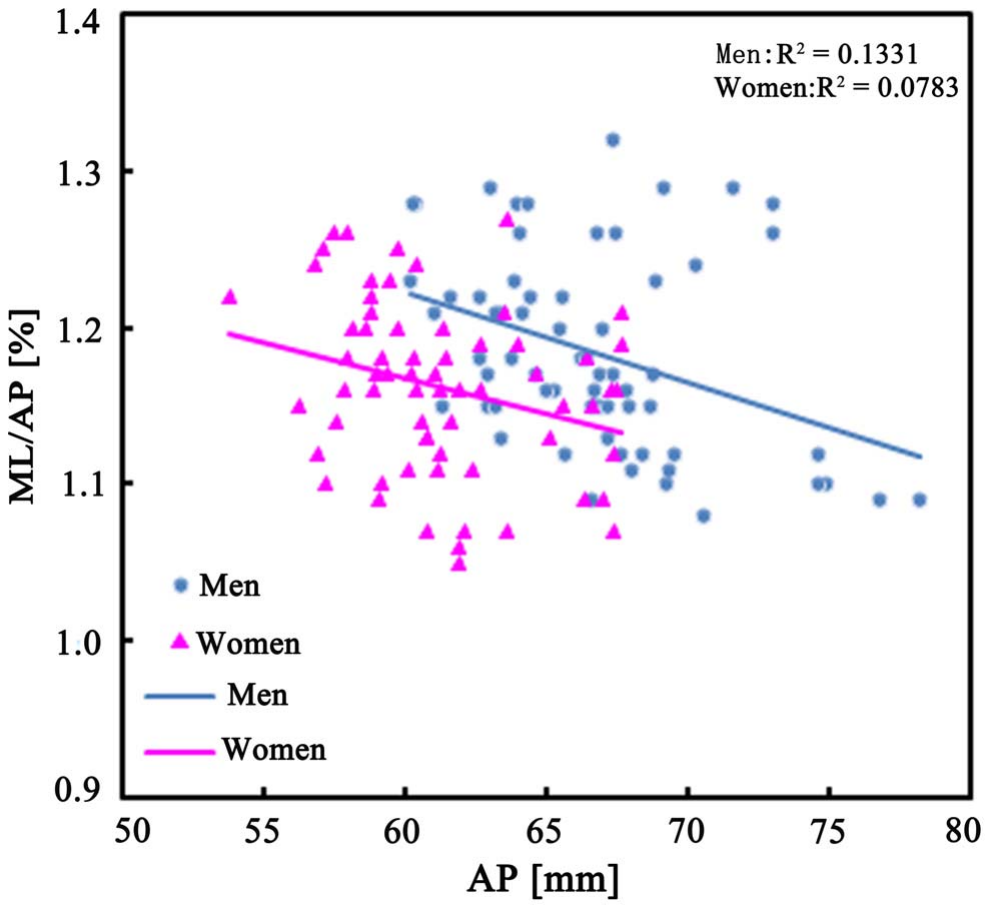
The ML/AP aspect ratios were plotted against the AP dimension for men and women.

The ML/AP aspect ratios for the distal femur showed a progressive decline with increasing AP dimension in both men and women. The fitted line for women lies below that of men, showing that women generally have smaller ML/AP ratios than men for a given AP dimension (Figure 3).

## Discussion

Researchers continue to debate whether it is necessary to design and use sex-specific femoral components based on anatomic differences in the morphology of the distal femur in men and women [8], [9]. Here we analyzed morphological differences in the distal femurs of 130 Chinese osteoarthritic knees based on simulated intraoperative bone cuts in three-dimensional CT images. The ALCH and the ML and AP dimensions of the resected distal femurs were significantly smaller in women than in men, while the AMCH values did not differ significantly according to sex. In addition, there was a significant sex difference in the ML/AP aspect ratio, with women generally having smaller ML/AP aspect ratios and narrower distal femurs than men.

Several researchers have studied the ML and AP dimensions of the distal femur in Asian populations. Lim et al. [20] used MRI to show that femoral ML and AP dimensions were 81.5±5.7 mm and 59.0±4.0 mm in men and 76.7±3.7 mm and 58.4±3.1 mm in women in a Korean population. Yue et al. [12] studied knees using CT imaging in a Chinese population and reported values of 82.6±3.6 mm and 65.0±2.8 mm in men and 72.8±2.6 mm and 58.8±2.5 mm in women. Notably, these studies looked at femur morphology in healthy knees. Such data may not suitable for guiding implant design because most knees undergoing TKA are deformed and sometimes differed dramatically from healthy knees. Instead, prosthetic designs should be based on data from diseased knees [18]. In this study, we measured osteoarthritic knees that were candidates for TKA; these data better reflect the morphology of patients seeking TKA and should thus be more useful for designing well-fitting components.

The difference in the distal femoral condyle ML/AP aspect ratio is one of the reasons for designing a sex-specific femoral implant. Our results and those of previous studies demonstrated that osteoarthritic women have narrower distal femurs and smaller ML/AP ratios than men [21], [5]. Hitt et al. measured 337 knees of TKA patients (209 women and 128 men) and found that with the use of standard femoral components there tended to be overhang in women compared to men [2]. Clarke et al. [7] reported that with standard femoral prostheses, the incidence of femoral component ML overhang in women was 17% compared with 0% in men. The overhang rate decreased in women when a female-specific femoral component was used. Guy et al. [6] measured the intraoperative anatomy of the distal femur in 100 knees of TKA patients (50 men and 50 women) and compared these measurements with the geometries of standard and female-specific femoral components. They found that with the use of standard femoral components, there was an overhang greater than 3 mm at the anterior flange width in 48% (24/50) of women and in the anterior medial-lateral width in 58% (29/50) of women. In contrast, only 6% (3/50) of women had any measurable medial-lateral component overhang greater than 2 mm when female-specific femoral implants were used. However, none of these studies quantified the clinical effects of the overhang.

Optimal design of femoral TKA components is critical for obtaining ideal coverage of the resected femur surface to allow for best function. We found that the distal femurs of women have smaller ML dimensions and aspect ratios than those of men for the same AP dimension. This means that for a prosthesis with a given AP dimension, the femoral ML dimension may overhang in women. Overhang can cause pain and soft tissue irritation, which is related to undesirable clinical outcomes [22]. Consequently, taking sex differences into consideration when designing sex-specific femoral components could decrease ML overhang and thereby achieve successful clinical outcomes.

Anterior condylar height is another issue that should be considered when designing sex-specific femoral components. Many studies have examined the sex differences of femoral morphology in Caucasians population. Poilvache et al. [21] measured TKA patients intraoperatively and found that the knees of women have smaller ALCHs and AMCHs than those of men. Conley et al. reported the similar results using CT imaging [3], [23]. Fehring et al. measured the anterior condylar height of men and women by MRI and found a significant difference in AMCH but no difference in ALCH [24]. Our results showed a significant difference between the sexes with regard to ALCH but not AMCH. Differences in different studies may be explained by anatomic variations according to ethnicity. Indeed, some studies have demonstrated differences in the shape and size of the knee in populations that differ in terms of ethnicity by three-dimensional CT or MRI images [25], [12]. In this study, we measured distal femur morphology of 130 Chinese osteoarthritic knees by three-dimensional CT images, and the morphology differed from that Caucasians. It is not known whether these differences are clinically relevant. Those in favor of sex-specific knee components posit that use of standard implants in women may overstuff the patellofemoral joint, leading to anterior knee pain and limited range of motion [3], [26], [8]. In contrast, others claim that the differences are small and not clinically important [27], [28]. Based on our study and the recent literature, sex differences in anterior condylar height do exist, but there is no clear clinical evidence concerning the effect of these differences on TKA.

Recent studies have reported substantial ethnic differences in distal femur morphology [12], [25]. In particular, Asian populations have been found to have smaller and narrower distal femurs than Caucasian populations [12], [13]. However, most TKA components that are currently in use were designed based on data from Caucasian populations. Some studies have looked at the fit of current femoral implant systems in Chinese, Korean, and Thai populations and found that implants tend to have overhangs in these Asian populations [1], [20], [29]. In terms of clinical results, Iorio et al. reported that Asian patients had significantly less postoperative range of motion and a higher revision rate after primary TKA than white patients [15]. These suggested ethnic differences should be taken into account when designing TKA components suitable for use in Asian populations.

One limitation of the present study is that we could not directly compare the morphology of the distal femur in Chinese and Caucasian populations. The depth of the distal femur resection affects the sizing of the resected surface. During the TKA procedures, the suitable depth of the distal femur resection varies on individual length of the femur. Another limitation is that the cutting depth in our study at a constant thickness of 9 mm above the lowest point of the medial femoral condyle. This should be noted when interpreting the results.

In conclusion, the present study found that Chinese women with osteoarthritic knees generally had smaller ALCHs and narrower ML dimension in their distal femurs compared to their male counterparts. The results suggested that in terms of prosthesis design, sex differences should be taken into account to design components that better match the natural geometry of femurs in men and women. Further study is needed to determine whether sex-specific component design leads to better clinical outcomes in TKA patients.
